# Counterclockwise rotation of the flagellum promotes biofilm initiation in *Helicobacter pylori*

**DOI:** 10.1128/mbio.00440-24

**Published:** 2024-05-03

**Authors:** Xiaolin Liu, Paphavee Lertsethtakarn, Vanessa T. Mariscal, Fitnat Yildiz, Karen M. Ottemann

**Affiliations:** 1 Department of Microbiology and Environmental Toxicology, University of California, Santa Cruz, California, USA; Georgia Institute of Technology, Atlanta, Georgia, USA

**Keywords:** chemotaxis, biofilm initiation, *H. pylori*, flagellar rotation

## Abstract

Motility promotes biofilm initiation during the early steps of this process: microbial surface association and attachment. Motility is controlled in part by chemotaxis signaling, so it seems reasonable that chemotaxis may also affect biofilm formation. There is a gap, however, in our understanding of the interactions between chemotaxis and biofilm formation, partly because most studies analyzed the phenotype of only a single chemotaxis signaling mutant, e.g., *cheA*. Here, we addressed the role of chemotaxis in biofilm formation using a full set of chemotaxis signaling mutants in *Helicobacter pylori*, a class I carcinogen that infects more than half the world’s population and forms biofilms. Using mutants that lack each chemotaxis signaling protein, we found that chemotaxis signaling affected the biofilm initiation stage, but not mature biofilm formation. Surprisingly, some chemotaxis mutants elevated biofilm initiation, while others inhibited it in a manner that was not tied to chemotaxis ability or ligand input. Instead, the biofilm phenotype correlated with flagellar rotational bias. Specifically, mutants with a counterclockwise bias promoted biofilm initiation, e.g., ∆*cheA*, ∆*cheW*, or ∆*cheV1*; in contrast, those with a clockwise bias inhibited it, e.g., ∆*cheZ*, ∆*chePep*, or ∆*cheV3*. We tested this correlation using a counterclockwise bias-locked flagellum, which induced biofilm formation independent of the chemotaxis system. These CCW flagella, however, were not sufficient to induce biofilm formation, suggesting there are downstream players. Overall, our work highlights the new finding that flagellar rotational direction promotes biofilm initiation, with the chemotaxis signaling system operating as one mechanism to control flagellar rotation.

**IMPORTANCE:**

Chemotaxis signaling systems have been reported to contribute to biofilm formation in many bacteria; however, how they regulate biofilm formation remains largely unknown. Chemotaxis systems are composed of many distinct kinds of proteins, but most previous work analyzed the biofilm effect of loss of only a few. Here, we explored chemotaxis’ role during biofilm formation in the human-associated pathogenic bacterium *Helicobacter pylori*. We found that chemotaxis proteins are involved in biofilm initiation in a manner that correlated with how they affected flagellar rotation. Biofilm initiation was high in mutants with counterclockwise (CCW) flagellar bias and low in those with clockwise bias. We supported the idea that a major driver of biofilm formation is flagellar rotational direction using a CCW-locked flagellar mutant, which stays CCW independent of chemotaxis input and showed elevated biofilm initiation. Our data suggest that CCW-rotating flagella, independent of chemotaxis inputs, are a biofilm-promoting signal.

## INTRODUCTION

Biofilms are multicellular communities of bacteria attached to biotic or abiotic surfaces. Bacteria in biofilms cause many environmental and therapeutic problems, as they are difficult to treat and detach. The human-associated gram-negative pathogen *Helicobacter pylori* has been reported to form biofilms (1–7). It lives in the microaerobic environment of the human stomach, and infection can cause acute and chronic gastritis, peptic ulcer disease, and gastric cancer (8, 9). *H. pylori* is classified as a class I carcinogen by the World Health Organization and infects about half of the population around the world (10). Antibiotic therapy is the major treatment for *H. pylori* infection (11); however, a quarter of infected individuals still remain uncured after using standard treatment (12, 13). Recently, experiments *in vitro* suggest that *H. pylori* forms biofilms that are tolerant to multiple antibiotics (14, 15). In microbes, biofilm formation occurs in three major steps: (i) initial attachment, (ii) aggregate development and maturation, and (iii) biofilm dispersion (16, 17). Several studies have analyzed *H. pylori* mature biofilms and identified proteins required for mature biofilm formation (4, 5, 7, 18–22). However, the signals and mechanisms that drive *H. pylori* to initiate biofilms remain unclear.

One important factor for biofilm initiation in many microbes, including *H. pylori*, is motility (20). *H. pylori* motility is propelled by lophotrichous flagella and enhanced by its helical cell shape (23). Similar to flagella reported in other species, *H. pylori* flagella are composed of more than 30 kinds of proteins, arranged in three major parts: motor, hook, and filament (24). At the base of the motor, flagella are powered by ion fluxes through the MotA and MotB stator complex. The *H. pylori* stator complex MotAB rings are surrounded by a cage-like structure, which is unique and different from the stator complex structure in other bacteria (25–27). This cage was recently identified to be composed of remote homologs of type IV pili proteins PilO, PilN, and PilM (28). *H. pylori* mutants that have defects in motility (∆*motB*) or flagella formation (∆*fliM,* ∆*fliA*) are poor biofilm formers at the mature stage, in part due to flagellar filaments in the biofilm matrix (4, 20). The flagellar cage proteins are also involved in biofilm formation, with mutants showing low biofilm mass only during early stages (28). These findings support that flagella and motility are important for *H. pylori* biofilm formation, but their role in early initiation is not fully understood.

Many bacteria, including *H. pylori*, achieve optimized migration by switching flagellar rotational direction between clockwise (CW) and counterclockwise (CCW). When *H. pylori* rotates its flagella in the CCW direction, the flagella lag behind the helical cell body and push cells forward (pusher mode) (29). In contrast, when *H. pylori* rotates its flagella in the CW direction, cells reverse their swimming direction frequently, and the flagella pull the cells (puller mode) (29, 30). The run-reverse swimming pattern is common for bacteria with lophotrichous flagella, e.g., *Pseudomonas putida, Vibrio fischeri,* and *Burkholderia* sp. *RPE64* (31). During the puller mode, *H. pylori* flagella at the leading pole may wrap around its helical cell body, as reported in other bacteria with lophotrichous flagella, e.g., *P. putida*, *Pseudomonas syncyanea*, and *V. fischeri*, or bacteria with bipolar flagella, e.g., *Campylobacter jejuni*, *Thiospirillum*, *Helicobacter suis*, and *Magnetospirillum magneticum* (31, 32). Overall, it is clear that *H. pylori* flagella rotate and confer motility in both directions.

The bacterial flagellar rotational direction is regulated in part by the chemotaxis system, which consists of a conserved set of proteins that monitor the environment and transduce these signals to control the flagella (33–35). The core sensing and signaling module is composed of chemoreceptors, the CheW coupling or scaffold protein, the CheA histidine kinase, and the CheY response regulator. In *Escherichia coli*, chemoreceptors, CheW, and CheA form a complex, with CheA histidine kinase activity regulated by chemoreceptor signal detection (36–38). CheA phosphorylates the response regulator CheY to CheY~P, which interacts with flagellar motor proteins to change the direction of motor rotation from the default CCW to CW. The mutation of *cheA*, *cheW,* or *cheY* results in the inability to create CheY~P and a flagellar motor that is CCW-biased. The CheY~P signal is terminated by a phosphatase, often CheZ, which accelerates the dephosphorylation of CheY~P. The mutation of *cheZ* results in high levels of CheY~P and CW-biased flagella. This set of reactions constitutes the chemotaxis signal activation pathway. In addition, there is a pathway that allows adaptation to sustained signals, regulated by the methyltransferase CheR and the methylesterase CheB. These proteins methylate or demethylate glutamyl residues on the chemoreceptors that in turn blunt or activate CheA, respectively. There are also other auxiliary chemotaxis proteins found in a subset of species such as CheV, CheX, CheC, CheD, FliY, ChePep, and CheS; these typically play roles as auxiliary scaffold or signal termination proteins (33).

*H. pylori* has a single chemotaxis pathway with many but not all components of the typical chemotaxis signaling pathway described above. *H. pylori* has four chemoreceptors (TlpA, TlpB, TlpC, and TlpD), CheA, CheW, and CheY as well as three CheV-type coupling/scaffold proteins named CheV1, CheV2, and CheV3 (39). *H. pylori* has the CheZ phosphatase and an additional chemotaxis protein, found only in Campylobacterota phylum members, called ChePep that has been shown to be important for CheZ polar localization and function (30, 40). *H. pylori* lacks the adaptation proteins CheR and CheB (41, 42). While motility and flagella are well established to play roles in *H. pylori* biofilm formation (20), there has been only one study that examined the role of chemotaxis in *H. pylori* biofilms (6). In this study, loss of *cheA* resulted in altered biofilms that were described as more flat and homogenous than wild-type (WT) biofilms, but with a greater percentage of the population adherent and present in the biofilm (6). This result supported the idea that chemotaxis plays a role in *H. pylori* biofilm formation and suggested that there may be increased biofilm associated with the loss of *cheA,* a phenotype that is unusual compared to *cheA* phenotypes reported for *E. coli* and other microbes (43–51).

In this work, we explored in more detail the roles the chemotaxis system plays during biofilm initiation and formation in *H. pylori*. In this study, we show that chemotaxis proteins affect biofilm initiation but are dispensable for mature biofilm formation. We find that some proteins promote and others inhibit biofilm initiation. The roles of chemotaxis proteins on biofilm formation were strongly correlated with their functions on flagellar rotation, such that CCW bias promoted biofilm initiation and CW bias inhibited it. According to these results, we propose that a major driver of biofilm formation is flagellar rotational direction, an idea we supported with CCW-locked *fliM* alleles, which are chemotaxis-independent. Finally, we collected data to show that CCW flagellar rotation activates but is not sufficient for biofilm initiation. Our results suggest that CCW-rotating flagella, independent of chemotaxis inputs, are a biofilm-promoting signal.

## RESULTS

### Chemotaxis proteins promote or inhibit the initial step of biofilm formation

**Fig 1:**
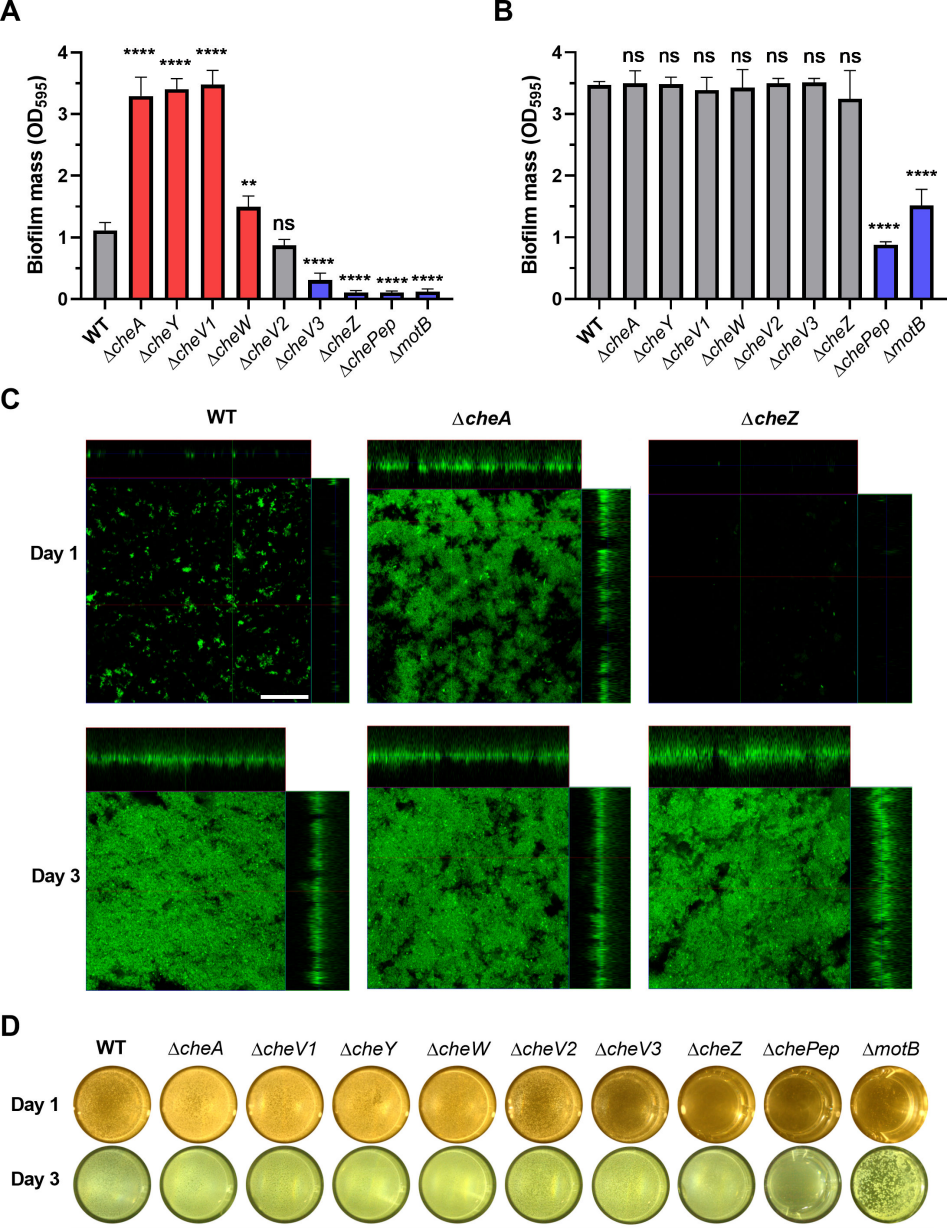
Biofilm formation by *H. pylori* G27 wild type and chemotaxis mutants. Biofilm mass formed on microtiter plate surfaces was quantified with crystal violet at optical density 595 (OD_595_) after culture for 1 day (**A**) or 3 days (**B**). Mutants with significantly increased or decreased biofilm mass compared to WT are colored in red or blue, respectively. (**C**) Representative images of GFP-expressing WT, Δ*cheA*, or Δ*cheZ* biofilms after culturing for 1 or 3 days. The large central panel is a top view, with the side panels representing a thin section of the attached cells or microcolonies that are taken at the indicated line. Scale bars = 50 µm. (**D**) Representative images of pellicles formed after 1 and 3 days. The background color difference is due to lighting on different days. In all panels, values shown are the mean ± standard deviation (SD) from at least three independent experiments. Statistical analyses were performed using one-way ANOVA (^****^P < 0.0001; ^**^P < 0.01), with asterisks indicating comparison to WT or NS for not significant.

To understand the role of chemotaxis in *H. pylori* biofilm formation, we first compared the biofilm formation of a ∆*cheA* mutant to an isogenic WT strain using 96-well static cultures, with plastic surfaces and crystal violet staining. After culturing for 24 hours, the ∆*cheA* mutant showed increased biofilm mass compared to WT (Fig. 1A), a finding that is similar to a previous study using glass surfaces (6). However, no significant differences were observed between ∆*cheA* and WT strain biofilm mass after 3 days, suggesting that CheA mostly acts at the initial biofilm steps (Fig. 1B). We then tested biofilm formation of mutants lacking each *H. pylori* chemotaxis signaling protein, including ∆*cheY*, ∆*cheW*, ∆*cheV1*, ∆*cheV2*, ∆*cheV3*, ∆*cheZ*, and ∆*chePep*. Some mutants, including ∆*cheV1*, ∆*cheY*, and ∆*cheW*, behaved the same as the ∆*cheA* strain: elevated biofilm mass compared to WT after 24 hours (Fig. 1A). In contrast, other mutants lacking *cheV3*, *cheZ*, or *chePep* resulted in low amounts of biofilm mass (Fig. 1A). There was no significant difference between the ∆*cheV2* mutant and WT (Fig. 1A). Although chemotaxis proteins played varied roles on biofilm formation after 1 day, the biofilm mass of all chemotaxis mutants, except ∆*chePep*, reached wild-type levels after culturing for 3 days (Fig. 1B). The non-motile strain ∆*motB* was used as a negative control, as it is known to exhibit a low biofilm-forming capacity (20). Overall, these results suggest that chemotaxis plays an important role in *H. pylori* biofilm initiation.

To further investigate the architectures and structures of *H. pylori* biofilms at different stages, we imaged the 1- and 3-day surface biofilms using confocal laser scanning microscopy of *H. pylori* WT and mutant strains expressing GFP. After culturing for 1 day, most WT cells on the surface were distributed as single cells, with some forming small aggregates (Fig. 1C). We then compared this phenotype to that of the ∆*cheA* strain, as a representative of the high-biofilm-forming phenotype, and the ∆*cheZ* strain, as a representative of the low-biofilm-forming phenotype. The biofilms of ∆*cheA* strains were composed of large amounts of adherent cells and aggregates, while the ∆*cheZ* mutant had only few attached single cells (Fig. 1C). After culturing for 3 days, WT formed mature and structured biofilms as previously reported (4, 20), as did the ∆*cheA* or ∆*cheZ* mutants (Fig. 1C). These results suggest that chemotaxis proteins mostly work at the initial stage of biofilm formation, and they either promote or inhibit this initial biofilm step.

Besides the biofilm formation on biotic or abiotic surfaces, *H. pylori* also forms a pellicle biofilm at the air–liquid interface (4, 52). We hypothesized that the surface biofilm phenotype would also be true for pellicle biofilms. We, therefore, tested the roles of different chemotaxis mutants on pellicle formation after 1 and 3 days. Consistent with the role of chemotaxis proteins on surface biofilm formation, after 1 day, the opacity of the pellicles formed by ∆*cheA*, ∆*cheV1*, ∆*cheY*, and ∆*cheW* strains was higher compared to that of WT, while ∆*cheV3*, ∆*cheZ*, and ∆*chePep* strains showed pellicles with low opacity (Fig. 1D), similar to the negative control strain ∆*motB*. There was no visible difference between WT and the ∆*cheV2* strain at 1 day (Fig. 1D). After 3 days, except for the ∆*chePep* strain, all mutants formed wild-type level pellicles, and ∆*motB* strain was used as a negative control (Fig. 1D). These results suggest that chemotaxis plays similar roles to speed up or slow surface and pellicle biofilms.

One possibility is that the WT-appearing biofilms at 3 days in the biofilm-defective mutants are the result of suppressor mutations. To examine this idea, we isolated single colonies from 4-day pellicle biofilms of the ∆*cheZ* strain and retested them for biofilm formation. These biofilm-isolated ∆*cheZ* strains were still defective in pellicle and biofilm formation compared to WT (Fig. S1), suggesting that the appearance of mature biofilms in these mutants is not due to the generation of genetic suppressors. Overall, these results suggest that chemotaxis proteins promote or inhibit biofilm formation in both surface and pellicle biofilms. Since the functions of chemotaxis proteins on biofilm and pellicle formation are similar, we focused our further studies on surface biofilms.

### Chemoreceptors work on biofilm initiation but independently of their ligands

**Fig 2:**
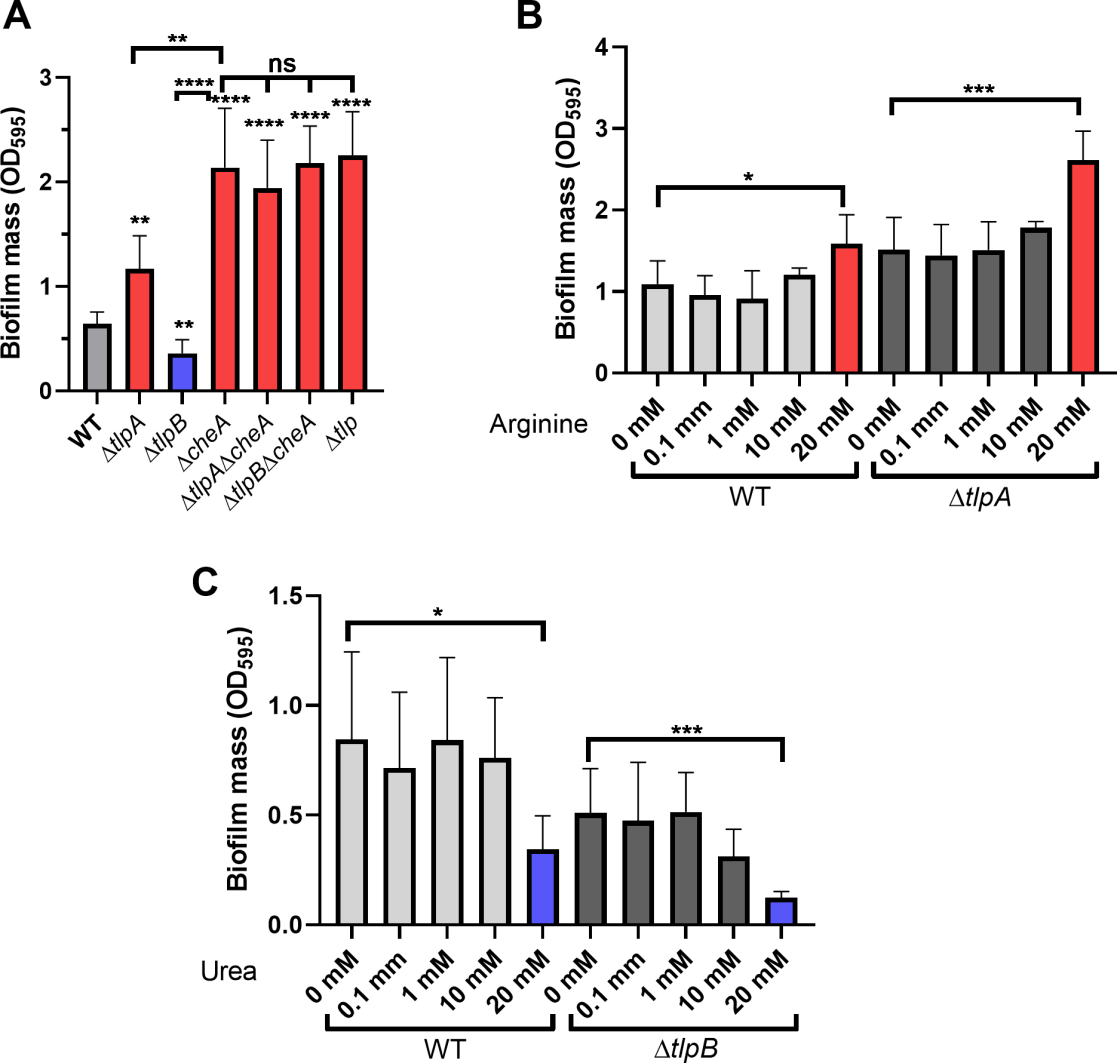
Biofilm formation by *H. pylori* G27 strains lacking the TlpA or TlpB chemoreceptors. Biofilm mass formed on microtiter plate surfaces was quantified with crystal violet at OD_595_ after culture for 1 day. Mutants or conditions with significantly increased or decreased biofilm mass compared to WT or untreated are colored in red or blue, respectively. (**A**) Biofilm formation by ∆*tlpA* or ∆*tlpB* mutants singly, combined with ∆*cheA* mutants, or mutants lacking all chemoreceptors (∆*tlp*). (**B**) Biofilm formation by WT and Δ*tlpA* mutant with different concentrations of the TlpA ligand arginine added throughout the biofilm formation assay period. (**C**) Biofilm formation by WT and Δ*tlpB* mutant with different concentrations of the TlpB ligand urea added throughout the biofilm formation assay period. All values are shown as the mean ± SDs from at least three independent experiments. Statistical analyses were performed using ANOVA (^*^P < 0.05; ^**^P < 0.01; ^***^P < 0.001; ^****^P < 0.0001; ns, not significant), with asterisks indicating comparison to WT (panel A) or untreated matched strains (panels B and C).

In addition to chemotaxis signaling proteins, we also tested the role of chemoreceptors on biofilm formation. If chemotaxis signaling operated during biofilm formation, we hypothesized that the chemoreceptor ligands would also affect biofilms. *H. pylori* has three transmembrane chemoreceptors—TlpA, TlpB, and TlpC—and one cytoplasmic chemoreceptor, TlpD (39). We focused on TlpA and TlpB because TlpC is not expressed in the G27 strain used in this study (53), and TlpD senses 'reactive oxygen species (ROS) but not chemical ligands (54). TlpA senses arginine, and TlpB senses urea (55–57). The ∆*tlpA* strain showed increased initial biofilm formation compared to WT, while the ∆*tlpB* one showed decreased initial biofilm formation (Fig. 2A). We then tested whether the TlpA and TlpB ligands would alter biofilm formation. In other microbes, e.g., *Comamonas testosteroni*, the addition of cognate ligands of chemoreceptors MCP2983 and MCP2201 triggered a significant increase of biofilm mass in a ligand and chemoreceptor-dependent manner (43). Arginine increased and urea decreased biofilm formation at high concentrations, 20 mM (Fig. 2B and C), and concentrations that are higher than the required amount, 10 mM, to induce a chemotaxis response (55, 56). Furthermore, these ligands acted independently of TlpA or TlpB (Fig. 2B and C), as the effects were observed in both WT and the deletion strains. This result suggested that chemoreceptors in *H. pylori* work on biofilm formation independently of their ligands. In support of this idea, *H. pylori* lacking all four chemoreceptors (∆*tlp*) showed high biomass that was similar to that observed with the ∆*cheA* strain (Fig. 2A). These results suggest that chemotaxis sensing is not needed for *H. pylori* biofilm initiation.

### The chemotaxis system, but not chemotaxis, is involved in biofilm initiation

**Fig 3:**
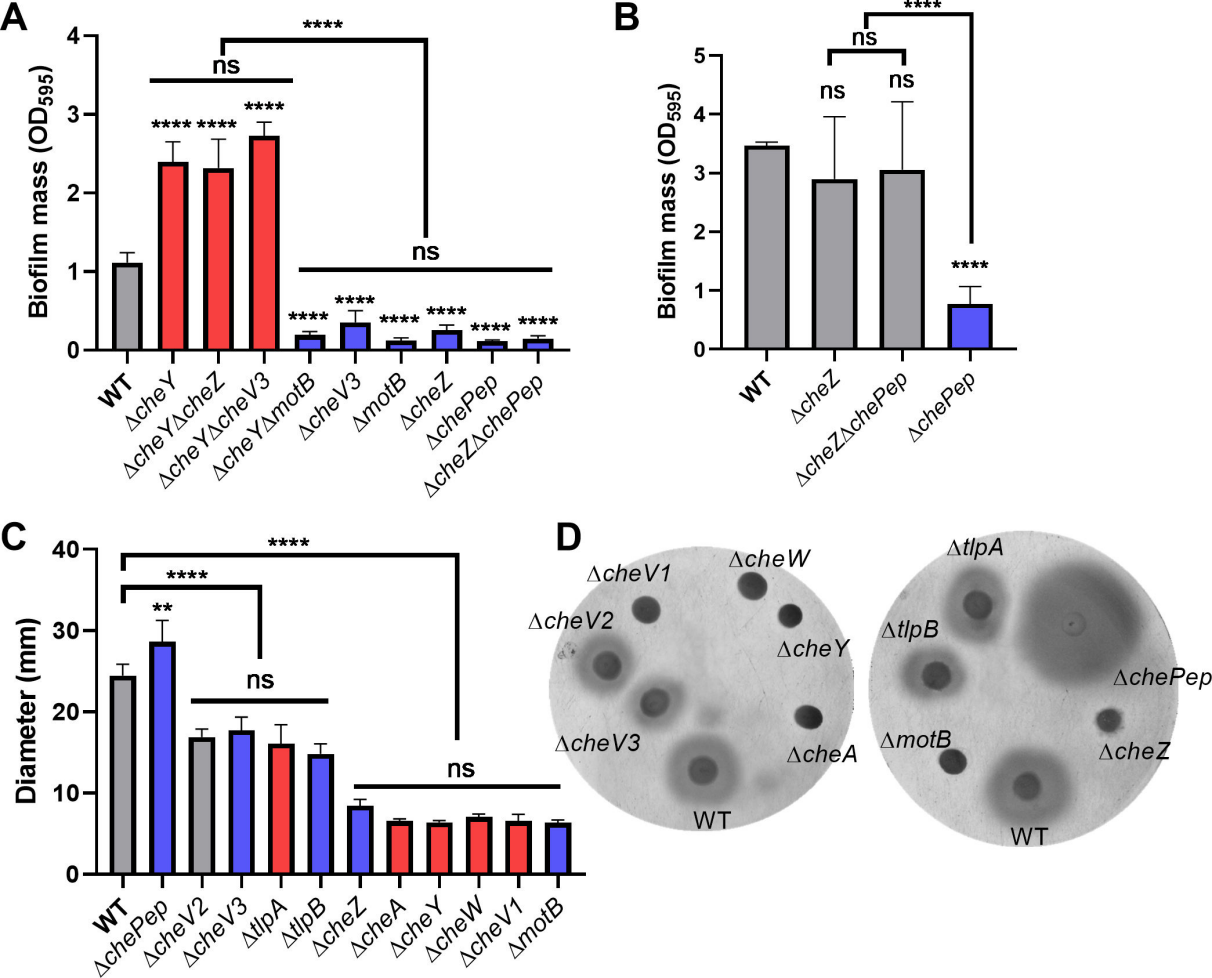
*H. pylori* G27 biofilm initiation is dependent on the chemotaxis pathway but not correlated with chemotaxis defects. Biofilm formation of different *H. pylori* G27 chemotaxis double mutants after 1 or 3 days. Biofilms were formed on 96-well plates and quantified with crystal violet as in Fig. 1 and 2. (**A**) Biofilm formation after culturing for 1 day. (**B**) Biofilm formation after culturing for 3 days. (**C**) Soft agar migration of WT and chemotaxis mutants in 0.35% soft agar after 4 days. Δ*motB* is non-motile and used as a negative control. (**D**) Representative soft agar images. In panels A–C, mutants showing increased or decreased biofilm mass compared to WT are colored in red and blue, respectively. Data are shown as the mean ± SDs from at least three independent experiments. Statistical analyses were performed using ANOVA (^**^P < 0.01; ^****^P < 0.0001), with asterisks indicating comparison to WT in each panel and ns indicating not significant.

The above results show that some chemotaxis proteins promote while others inhibit biofilm initiation. We, therefore, asked whether these proteins operate in the same pathway as each other, versus in different pathways. To test this notion, we constructed double mutants and analyzed their epistatic relationships. For signal input, we constructed ∆*cheA*∆*tlpA* and ∆*cheA*∆*tlpB* double mutants, and both exhibited similar biofilm mass as ∆*cheA*, suggesting that CheA is downstream of TlpA and TlpB in the biofilm pathway (Fig. 2A). Because CheY is the most downstream component in the chemotaxis pathway and shows opposite roles in biofilm initiation compared to ∆*cheV3* and ∆*cheZ*, we constructed ∆*cheY*∆*cheV3* and ∆*cheY*∆*cheZ* to analyze their epistatic relationships. The ∆*cheY*∆*cheV3* and ∆*cheY*∆*cheZ* both showed similar phenotype as ∆*cheY*, suggesting that CheY acts downstream of CheZ and CheV3 in initial biofilm formation (Fig. 3A). ∆*chePep* was the sole chemotaxis mutant that showed a defect on biofilm mass after culturing for 3 days (Fig. 1B). Previous studies indicated that ChePep helps recruit CheZ to the cell poles (30). The double mutant ∆*cheZ*∆*chePep* had a similar biofilm mass as WT and ∆*cheZ* after culturing for 3 days (Fig. 3B), indicating that CheZ works downstream of ChePep on biofilm formation. These results suggest that the pathway leading to biofilm formation operates in basically the same order as that controlling chemotaxis, with CheY acting most downstream.

Because chemotaxis proteins play varying roles in biofilm formation, we examined whether there was a correlation with their roles in chemotaxis ability in a standard chemotaxis assay, soft agar migration (58). If chemotaxis signaling operated similarly in both biofilm initiation and chemotaxis, we hypothesized that the extent of defects would correlate between the two. We, however, did not detect a correlation. For example, ∆*cheV3* and ∆*tlpA* both have minimal soft agar migration defects (Fig. 3C and D) (59–61) but caused opposite effects (reduced or enhanced) on biofilm initiation (Fig. 1A and 2A). ∆*cheZ* and ∆*cheA* both caused equally significant migration defects (Fig. 3C), but opposite biofilm phenotypes (Fig. 1). The deletion of ∆*tlpA* and ∆*tlpB* showed subtly lower soft agar migration (Fig. 3C and D) (54, 62); however, ∆*tlpB* showed decreased biofilm, while ∆*tlpA* showed increased biofilm (Fig. 2). These results suggest that the divergent function of chemotaxis proteins on biofilm formation cannot be explained by chemotaxis behavior.

### Phosphorylation of CheY is involved in biofilm initiation

**Fig 4:**
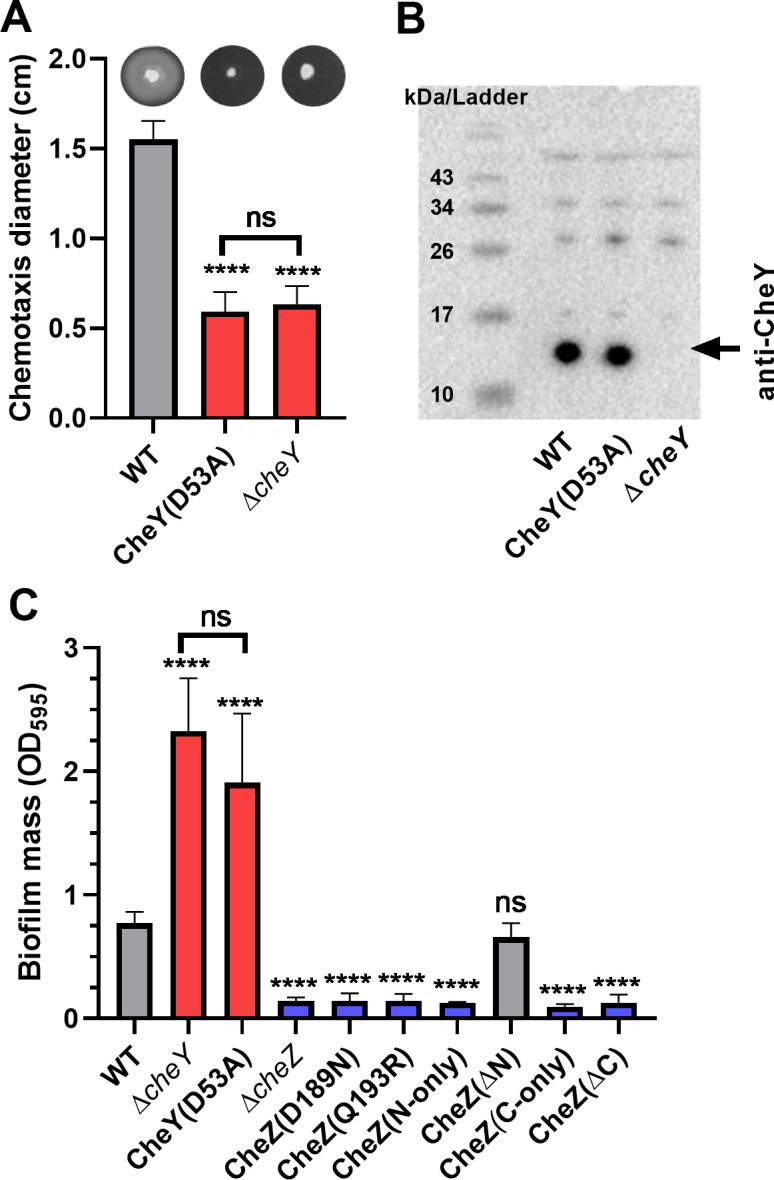
CheY phosphorylation is required for normal chemotaxis and biofilm formation. (**A**) Chemotaxis migration (colony diameter) in 0.35% soft agar plates after 3 days. Representative images of the halo formed by cognate strains in soft agar plates are shown at the top of panel A. (**B**) Western blot analysis using Anti-CheY antibody on 15% SDS-PAGE gels. Molecular weight markers are shown in the left lane of panel B. The arrow indicates the position of CheY, at the predicted molecular weight of 13.9 kD. (**C**) Biofilm formation of *cheY* and *cheZ* mutants on 96-well plates after culturing for 1 day. Biofilm mass was determined using crystal violet staining as in Fig. 1–3. In panels A and C, mutants showing significantly increased or decreased biofilm mass compared to WT were colored in red and blue, respectively. Data shown are the means ± SDs from at least three independent experiments. Statistical analyses were performed using ANOVA (^****^P < 0.0001), with asterisks indicating comparison to WT in each panel and ns indicating not significant.

Given the findings above that CheY is the most downstream in the biofilm-controlling pathway, we next asked whether CheY phosphorylation was essential for the role of CheY in biofilm formation. D53 is the key site of CheY phosphorylation (63, 64), so we generated a CheY(D53A) site-directed mutant and introduced it back into the *H. pylori cheY* locus as the sole *cheY*. As this mutation has not been characterized in *H. pylori*, we confirmed that the mutant with CheY(D53A) showed a severe defect on soft agar migration, equivalent to a *cheY* null mutant (Fig. 4A) but is expressed to normal levels (Fig. 4B). We then tested the biofilm formation of the strain with CheY(D53A) and found that it showed increased biofilm mass after culturing for 1 day, similar to the ∆*cheY* mutant (Fig. 4C). These results suggest that phosphorylation of CheY is critical for biofilm initiation.

We provided further evidence for the role of CheY phosphorylation by turning to the CheY phosphatase, CheZ. The CheZ active site relies on D189 and Q193 for dephosphorylating CheY~P (63), so we queried how the CheZ(D189N) and CheZ(Q193R) mutants would affect biofilm initiation. Strains bearing these *cheZ* alleles showed low biofilm mass that was not different from the ∆*cheZ* mutant, suggesting that dephosphorylating activity of CheZ is required for the biofilm initiation phenotype (Fig. 4C; Fig. S2). We also queried the roles of the CheZ N-terminal region, which has an unknown function, by testing different truncation mutants that lack N-terminal regions of CheZ (63). Previous work had shown that deleting the N-terminal region does not affect CheZ phosphatase function, while deleting the C-terminal region does (63). Similar results were found with biofilm formation: deleting the N-terminal regions did not affect the role of CheZ in biofilm formation (Fig. 4C; Fig. S2). However, if we used *cheZ* mutants that abolished phosphatase activity, due to the deletion of regions at the C-terminus or retention of only the N-terminal or C-terminal regions, all showed severe defect on biofilm formation (Fig. 4C). Taken together, these results provide strong support for the idea that CheY phosphorylation is important for its ability to promote biofilm initiation.

### The motor rotational direction correlates with biofilm initiation ability

**Fig 5:**
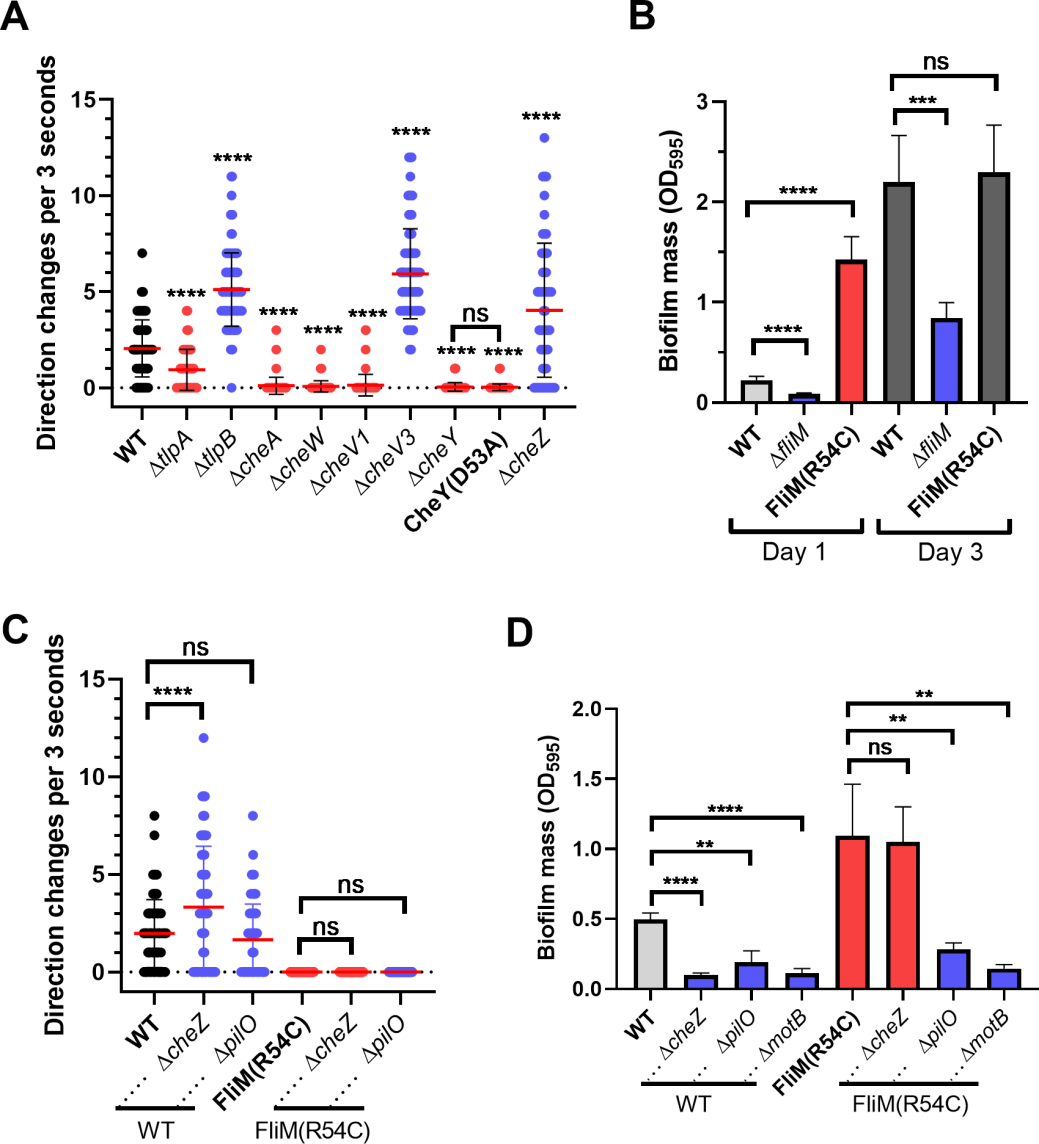
Chemotaxis reversal frequency correlates with biofilm-forming ability. (**A**) The reversal frequency of *H. pylori* G27 chemotaxis mutants was determined by the quantification of reversals in swimming cultures in BB10 media. For each strain, 57–98 cells were quantified. (**B**) Biofilm formation of CCW-locked *H. pylori* strains NSH57 [FliM (R54C)], ∆*fliM* mutant LSH99, and WT LSH100 after 1 and 3 days. (**C**) Changes of direction during swimming in BB10 media were quantified by tracking the behavior of individual cells. For each strain, 58–116 cells were quantified. (**D**) Biofilm formation of Δ*cheZ* and Δ*pilO* deleted in the WT (LSH100) or FliM (R54C) (NSH57) backgrounds after culturing for 1 day. Biofilms were formed and analyzed as in Fig. 1. In each panel, mutants showing increased or decreased biofilm mass compared to WT were colored in red and blue, respectively. Data shown are the means ± SDs from at least three independent experiments. Statistical analyses were performed using ANOVA (^**^P < 0.01; ^***^P < 0.001; ^****^P < 0.0001), with asterisks indicating comparison to WT and ns indicating not significant.

Our results above showed that some chemotaxis mutants exhibit increased or decreased initial biofilm mass, in a manner that is dependent on CheY phosphorylation. In *H. pylori*, like many bacteria, chemotaxis proteins affect chemotaxis behavior by regulating the flagellar rotational direction switching. We, therefore, examined whether there was a correlation between how the chemotaxis signaling mutants affect flagellar rotation and their effect on biofilm formation. Flagellar rotational direction can be inferred by microscopically examining bacterial swimming behavior (29). Mutants that swim with few reversals have a CCW bias, including ∆*cheW*, ∆*cheA*, and ∆*cheY* mutants (54, 60, 65). In contrast, mutants that reverse frequently, including ∆*cheZ*, ∆*chePep*, and ∆*cheV3,* have been reported to have a CW bias (30, 40, 60). We, therefore, evaluated the frequency of directional reversals of these mutant strains using microscopy, confirming the phenotypes of the mutants above as well as others tested here (Fig. 5A). Mutants having a high reversal frequency and, therefore, CW bias were poor biofilm initiators, including ∆*cheZ*, ∆*chePep*, ∆*cheV3*, and ∆*tlpB* (Fig. 5A; Table 1). In contrast, mutants with few reversals and, therefore, CCW bias showed high biofilm initiation, including ∆*cheA*, ∆*cheW*, ∆*cheV1*, ∆*cheY*, and ∆*tlpA* (Fig. 5A; Table 1). These observations, combined with the lack of responsiveness to chemotaxis ligands (Fig. 3C), suggested that the role of chemotaxis proteins on biofilm initiation might be achieved by affecting flagellar rotational direction rather than chemotaxis *per se*. Our data suggested that CCW-rotating flagella place *H. pylori* into a biofilm-promoting state. In contrast, if the flagella rotate CW, *H. pylori* biofilm initiation was inhibited.

**TABLE 1:** Phenotypes of *H. pylori* chemotaxis mutants compared to WT for biofilm formation, swimming trajectory, and chemotaxis ability on soft agar plates

Mutants	Biofilm mass	Smooth/CCW or reversal/CW bias		Diameter of halo on soft agar plates	
∆*tlpA*	Increased^*a*^	Smooth	Fig. S1	Subtle difference	Fig. 3C and D (54)
∆*tlpB*	**Decreased^*b*^**	**Reversal**	Fig. S1	Subtle difference	Fig. 3C and D (54)
∆*cheA*	Increased	Smooth	Fig. 5A (54)	Severe decrease	Fig. 3C and D (54)
∆*cheY*	Increased	Smooth	Fig. 5A (60)	Severe decrease	Fig. 3C and D (60)
∆*cheW*	Increased	Smooth	Fig. 5A (65)	Severe decrease	Fig. 3C and D (60)
∆*cheZ*	**Decreased**	**Reversal**	Fig. 5A (30)	Severe decrease	Fig. 3C and D
∆*chePep*	**Decreased**	**Reversal**	(40)	Severe decrease	Fig. 3C and D
∆*cheV1*	Increased	Smooth	Fig. 5A (60)	Severe decrease	Fig. 3C and D (60)
∆*cheV3*	**Decreased**	**Reversal**	Fig. 5A (60)	Subtle difference	Fig. 3C and D (60)
CheYD53A	Increased	Smooth	Fig. 5A	Severe decrease	Fig. 4A

^*a*^ The matches of increased biofilm mass and smooth swimming behavior are indicated with underlined type.

^*b*^ The matches of decreased biofilm mass and reversal swimming behavior are indicated with bold type.

### CCW flagellar motor rotation is important but not sufficient to initiate biofilm formation

The above results suggest that *H. pylori* biofilm initiation may be the consequence of flagellar rotational direction. To directly test this idea, we made use of a previously characterized *fliM* allele that results in locked CCW flagellar rotational behavior independent of chemotaxis inputs, by changing FliM amino acid 54 from arginine to cysteine [FliM (R54C)] (66). This strain displays few reversals, consistent with CCW bias as reported previously (Fig. 5C). The CCW-biased FliM (R54C) strain formed a significantly higher biofilm mass than its isogenic WT after 1 day (Fig. 5B). In contrast, the ∆*fliM* null mutant was non-motile and did not initiate biofilm formation (Fig. 5B). This finding supported the idea that CCW flagellar bias strongly promoted biofilm initiation. We then tested whether CCW-biased flagellar rotation was sufficient to induce biofilm independently of chemotaxis. We combined FliM (R54C) with a ∆*cheZ* mutant, to create a strain with high intracellular levels of CheY~P (63) but a CCW-locked swimming behavior (Fig. 5C). This strain formed high levels of biofilm (Fig. 5D), showing that flagellar rotation overrode the high CheY~P signal that normally inhibited biofilm formation (Fig. 1A). This result furthermore suggested that biofilm initiation did not require chemotaxis signaling from CheZ. The FliM (R54C) mutation did not confer increased biofilm formation when combined with ∆*motB* (Fig. 5D). This strain is predicted to be flagellated but non-motile (20, 67), indicating rotating flagella are needed for the biofilm initiation response. These results are consistent with a model in which CCW flagellar rotation is a strong signal to induce biofilm initiation, and flagellar rotation activates biofilm initiation by a route that does not rely on the chemotaxis signaling proteins.

We further asked whether CCW rotation was sufficient to cause biofilm initiation. In *E. coli*, CCW-biased swimming cells also have elevated biofilm initiation, because they are prone to move near surfaces and attach at a high rate (68). In this case, CCW rotation creates cells with elevated surface interactions. If this idea extends to *H. pylori*, we would hypothesize that CCW-rotating flagella should override mutations with low biofilm-forming initiation, provided they do not have defects in adherence or biofilm formation. One such mutant was recently identified that arose from the loss of the flagellar cage protein PilO/HPG27_252, located in the inner membrane near MotAB (28). This ∆*pilO* mutant exhibits decreased initial biofilm mass (Fig. 5D), normal mature biofilm formation, and normal directional flagella rotations (Fig. 5C) (28). We, therefore, combined the CCW-biased FliM (R54C) strain with the ∆*pilO* strain. This mutant retained the strong CCW bias phenotype of the FliM (R54C) (Fig. 5C) but exhibited the reduced biofilm initiation phenotype similar to the ∆*pilO* strain (Fig. 5D). These findings suggest that CCW rotation is a strong driver of biofilm initiation, but not sufficient to promote the process in all contexts.

## DISCUSSION

Biofilm growth provides many advantages, including access to nutrients, protection under complex environments, and the ability to survive challenges from compounds like antibiotics (69, 70). *H. pylori* has been documented in multiple studies to form a biofilm, but the early initiation steps have not been investigated thoroughly. Here, we report that an early signal for biofilm formation comes from flagellar rotation, an ability influenced by chemotaxis signaling. Indeed, our work shows for the first time that CCW flagellar rotation enhances early biofilm formation, while CW rotation inhibits it.

We happened upon the idea that flagellar rotational direction is a driver of biofilm initiation from work on chemotaxis signaling proteins. Our findings support the idea that chemotaxis is not essential for biofilm formation, and instead, the rotating flagellum is the dominant biofilm-ON signal. We reached the conclusion that chemotaxis *per se* is not important using a broad set of CCW- and CW-biased mutants, chemotaxis ligand addition, and the use of *cheZ* mutants in the context of locked CCW-biased flagella. However, our data are not necessarily inconsistent with previous work that chemotaxis and motility play important roles in biofilm formation (16, 17, 71). It remains possible that the chemotaxis response may contribute to biofilm formation, for the following reasons: (i) we used chemotaxis mutants with extreme CCW- or CW-biased flagellar rotation, (ii) we did not explore whether the switching between CCW/CW rotation affects biofilm formation, (iii) we did not test all chemotaxis ligands, and (iv) the attachment efficiency of single cells may be masked by the level of an averaged cell population.

One idea consistent with our data is that *H. pylori* flagellar rotation may represent a mechanical signal to affect biofilm initiation. Mechanical stress is known to occur on the flagella when bacteria transfer from swimming in a bulk fluid to attaching to the surface (72). Studies in *E. coli* showed that CCW-biased swimming cells are prone to move near a glass surface and then attach to surfaces at a higher rate than tumble-swimming cells (CW bias) (68). In this case, CCW bias creates cells that are more biofilm-prone. We, thus, hypothesized that CCW bias would be sufficient for *H. pylori* biofilm initiation, but this was not the case. We directly tested this idea using the FliM (R54C) ∆*pilO* double mutant. This strain retains CCW bias but does not enhance biofilm initiation. Thus, it seems that in *H. pylori,* CCW rotation may not simply drive the bacteria to the surface and facilitate contact.

Our data are consistent with the possibility that flagellar rotation could activate a downstream signaling pathway that promotes biofilm initiation. In other cases of flagellar-based mechanical signaling, increased numbers of *E. coli* stators (MotA and MotB) were recruited to the flagella to produce more torque in response to high load (73). In *H. pylori*, the WT flagella is fully occupied with stators (25, 28), suggesting there may be less of a range of MotAB numbers at the flagella, and this form of mechanosensing may not operate. There is a gap, however, in our understanding of how flagella-related mechanosignaling may operate in *H. pylori*. The *H. pylori* flagella is surrounded by a unique cage structure, which was recently identified to be composed of homologs of type IV pili PilM, PilN, and PilO (28, 74). Liu et al. suggest that PilM/N/O are involved in a surface response that consists of repressing motility and enhancing biofilm initiation, but the PilM/N/O proteins do not appear to sense surface signals directly because they are not at the bacterial or flagellar surface (28). The localization of PilM, PilN, and PilO also suggests that they do not act directly as biofilm matrix or adhesion factors. Loss of PilO creates strains that have low biofilm initiation in WT and CCW-biased backgrounds. These data suggest that CCW bias does not create some type of surface interaction that drives biofilm formation in all contexts, as suggested in *E. coli* where CCW rotation increases adherence in some conditions (68), and instead, the CCW signal depends on at least PilO for biofilm activation.

Here, we show that flagellar rotation is a key signal regulating biofilm formation in *H. pylori*, but whether this idea holds true in other microbes remains to be determined. As mentioned above, studies in *E. coli* reported that CCW-biased mutants adhered better under some conditions, while CW-biased mutants adhered poorly (68). Most studies on chemotaxis in other microbes have employed only single chemotaxis pathway mutants. Interestingly, most studies utilized *cheA* mutations, and these mutants generally formed less biofilm, opposite to the phenotype observed in *H. pylori*. There are hints, however, that there may be differential biofilm stimulation between CW and CCW flagellar rotation, e.g., in *Bacillus subtilis* and *Azorhizobium caulinodans* (50, 51). In *B. subtilis*, CW-biased mutants ∆*cheA* and ∆*cheY* exhibit less biofilm formation when competed against the wild-type strain, while CCW-biased ∆*cheC* and ∆*cheB* mutants, with the opposite flagellar rotational direction compared to *cheA* and *cheY* mutants, outcompete WT in a biofilm assay (50). In *A. caulinodans*, CCW-biased ∆*cheZ* has the opposite effect on CW-biased *cheA* mutants, with higher biofilm mass and higher extracellular polysaccharides than WT (51, 75). These studies suggest that flagellar rotational direction may be a widespread biofilm initiation signal in bacteria.

In sum, our data support the idea that CCW rotation promotes *H. pylori* biofilm initiation while CW rotation, in contrast, results in low biofilm initiation. We present data that flagella rotation is a dominant signal for this behavior, substantially controlled by the chemotaxis system, but that the CCW rotation is not solely able to drive biofilm initiation: in mutants lacking *pilO*, CCW-rotating flagella do not enhance biofilm initiation. This finding supports that the CCW rotation *per se* does not create better adherence. One possibility is that flagellar rotation operates in a mechanical signaling pathway that relies on PilO and likely other proteins. Unlike most other bacteria, *H. pylori* lacks the biofilm-related second messenger c-di-GMP (76), so any signaling output appears to be unrelated to that second messenger. Thus, our results show that CCW rotation is a strong biofilm-ON signal that can be driven by the chemotaxis system but that requires additional cell proteins to create the biofilm-initiating state in an as-yet-to-be-determined mechanism.

## MATERIALS AND METHODS

### Bacterial strain and growth conditions

*H. pylori* WT and its derivative strains used in this study are listed in Table 2. *H. pylori* was cultured on Columbia horse blood agar (CHBA) (Difco), with 0.2% β-cyclodextrin, 10 µg/mL vancomycin, 5 µg/mL cefsulodin, 2.5 U/mg polymyxin B, 5 µg/mL trimethoprim, and 8 µg/mL amphotericin B (all chemicals from Thermo Fisher or Gold Biotech). Liquid culture was carried out using Brucella Broth (BD BBL/Fisher) containing 10% heat-inactivated fetal bovine serum (FBS) [BB10 (Gibco/BRL)]. Both cultures on plates and in liquid were grown under microaerobic conditions (5% O_2_, 10% CO_2_, and 85% N_2_) at 37°C. For mutant selection, CHBA plates with 25 µg/mL of chloramphenicol or 75 µg/mL of kanamycin were used.

**TABLE 2:** Strains were used in this study

*H. pylori* strain		Genotype or description	Reference and/or source(s)
G27		Wild type	(77)/from Nina Salama
G27-GFP		G27 pTM115-GFP	(78)
mG27		G27, mouse-adapted	(79)
LSH100	KO1275	G27 NSH57 ∆*fliM*::*cat-sacB* with restored *fliM*	(66)/from Nina Salama
∆*cheA*	KO857	G27 ∆*cheA*::*cat* (also called ∆*cheAY*::*cat*)	(30)
∆*cheW*	KO851	G27 ∆*cheW*::*aphA3*	(30)
∆*cheY*	KO771	G27 ∆*cheY*::*cat*	This study
∆*cheY*	KO1250	G27 ∆*cheY*::*aphA3*/*sacB*	This study
∆*cheZ*	KO1269	G27 ∆*cheZ*::*aphA3*/*sacB*	
∆*chePep*	KO1332	G27-MA Δ*chePep*::*cat*	(40)
∆*cheV1*	KO1277	G27 ∆*cheV1*::*cat*	(30)
∆*cheV2*	KO1278	G27 ∆*cheV2*::*cat*	(30)
∆*cheV3*	KO1279	G27 ∆*cheV3*::*cat*	(30)
∆*tlpA*	KO1002	mG27∆*tlpA*	(80)
∆*tlpB*	KO1004	mG27 ∆*tlpB*	(80)
Δ*motB*	KO489	G27 Δ*motB*::*aphA3-sacB*	(67)
∆*tlpA*∆*cheA*	KO1770	mG27 ∆*tlpA*∆*cheA*::*cat*	This study
∆*tlpB*∆*cheA*	KO1771	mG27 ∆*tlpB*∆*cheA*::*cat*	This study
∆*tlp*	KO1021	mG27 ∆*tlpA*∆*tlpB*∆*tlpC*::*aphA3*∆*tlpD*::*cat*	(54)
∆*chePep*∆*cheZ*	KO1337	G27 ∆*cheZ*::*aphA3*/*sacB*∆*cheZ*::*cat*	This study
∆*cheY*∆*cheZ*	KO1772	G27 ∆*cheZ*∆*cheY*::*cat*	This study
∆*cheY*∆*cheV3*	KO1773	G27 ∆*cheY*∆*cheV3*::*cat*	This study
∆*cheY*∆*motB*	KO1774	G27 ∆*cheY*∆*motB*::*aphA3-sacB*	This study
∆*cheZ*::*cheZ*D189N	KO1036	G27 ∆*cheZ*::*cheZ*D189N	(30)
∆*cheZ*::*cheZ*Q193R	KO1037	G27 ∆*cheZ*::*cheZ*Q193R	(30)
*cheZ* N-only	KO1273	G27 ∆*cheZ*::*cheZ* 1–39 (retains amino acids 1–39)	(30)
*cheZ* C-only	KO1312	G27 ∆*cheZ*::*cheZ* C-only (retains amino acids 241–253)	(30)
*cheZ* ΔN	KO1313	G27 ∆*cheZ*::*cheZ*ΔN (deletion of amino acids 1–39)	(30)
*cheZ* ΔC	KO1300	G27 ∆*cheZ*::*cheZ*ΔC (deletion of C-terminal 12 amino acids)	(30)
CheY(D53A)	KO1775	G27 ∆*cheY*::*cheY*(D53A)	This study
LSH99	KO1776	G27 NSH57 ∆*fliM*::*cat-sacB*	(66)/N. Salama
LSH100∆*cheZ*	KO1777	LSH100 ∆*cheZ*::*aphA3*	This study
LSH100∆*pilO*	KO1778	LSH100 ∆*pilO*::*aphA3*	This study
NSH57	KO1779	G27 mouse-adapted G27 with a R54C substitution in FliM	(66)/N. Salama
NSH57∆*cheZ*	KO1780	NSH57 ∆*cheZ*::*cat*	This study
NSH57∆*pilO*	KO1781	NSH57 ∆*pilO*::*aphA3*	This study

### Design and construction of mutants

All mutants were constructed by natural transformation, as described previously (80). Single mutants were constructed by transforming WT G27 with the indicated plasmids, and double mutants were constructed by transforming the indicated starting strains with the indicated plasmids. The plasmid pKO127 previously used to delete *cheY* in SS1 strain (81) was used to delete *cheY* in G27 strain WT and ∆*cheZ* mutant in this study, resulting in ∆*cheY* and ∆*cheZ*∆*cheY*. To construct ∆*tlpA*∆*cheA* and ∆*tlpB*∆*cheA* double mutants, the plasmid pKT22 (30) was used to delete *cheA* in ∆*tlpA* and ∆*tlpA* backgrounds. To construct ∆*cheA*∆*cheW*, ∆*cheV1*∆*cheW*, and ∆*cheY*∆*cheW* double mutants, the plasmid pKT11 (30) used for *cheW* deletion in SS1 strain was used to delete *cheW* in ∆*cheA*, ∆*cheV1*, and ∆*cheY*. To construct ∆*chePep*∆*cheZ* and ∆*cheW*∆*cheZ*, the plasmid pKT31 used for deleting *cheZ* in SS1 strain (65) was used to delete *cheZ* in ∆*chePep* and ∆*cheW* mutants. To construct ∆*cheY*∆*cheV3*, the *cheV3::cat* and its neighboring upstream and downstream sequences were amplified from the genome DNA of *cheV3::cat* (30) with the cognate primers. The amplicon was introduced into *H. pylori* G27 with natural transformation. The plasmid pKO114i (67) was used to delete *motB* in ∆*cheY* mutant backgrounds. The positive colonies were screened using CHBA plates with specific antibiotics and confirmed by sequencing. The plasmid pKO126 (30) containing full-length *cheY* was used to carry out site-directed mutagenesis to create the D53A variant using inverse PCR. The resulting plasmids were introduced into the ∆*cheY::aphA3/sacB* strain to construct CheY site mutants. CHBA plates with 12% sucrose were used to select sucrose-resistant colonies, which were then screened for loss of kanamycin sensitivity. The positive colonies were further confirmed by PCR and sequencing.

### Biofilm formation on abiotic surfaces

The BB10 medium was used to culture *H. pylori* strains overnight. The next day, the OD_600_ of cells was adjusted to 0.15 with fresh BB10, and then, 0.2 mL was used to inoculate a sterile 96-well polystyrene microtiter plate (Costar no. 3596). Plates were incubated under standard *H. pylori* conditions with no shaking for 1 or 3 days. For biofilm mass quantification, the planktonic cells were removed by pipetting, and the wells were washed with 0.3 mL of sterile phosphate-buffered saline (PBS) twice. Three hundred microliters of crystal violet (0.1% [wt/vol]) was then added to the wells and incubated for 10 min at room temperature to stain the biofilm. After staining, the wells were washed twice with PBS, and the crystal violet stain was solubilized with 200 µL of ethanol (70% [vol/vol]). The biofilm mass of each well was quantified according to the absorbance at 590 nm.

### Confocal laser scanning microscopy

Cells for biofilm formation were prepared as described above using BB10. After being adjusted to OD_600_ of 0.15 with fresh BB10, 300-µL liquid cultures were added into each well of μ-Slide 8-well glass bottom chamber slides (ibidi, Germany) and a cover slip that was placed vertically in each well. The slides were placed into an incubator under microaerobic conditions (5% O_2_, 10% CO_2_, and 85% N_2_) at 37°C for 1 or 3 days. The pellicles at the air–liquid surface and the medium were removed, and the well was washed with PBS at least three times to remove unattached cells. After washing, 400 µL PBS was added to the well, and the image of biofilm on the cover slip was captured using a Zeiss 880 confocal microscope with a 488-nm laser.

### Western blotting

All overnight cell cultures were adjusted to OD_600_ of 0.7. Samples were lysed by heat (100°C) for 10 minutes and then run on 15% SDS-polyacrylamide gel with beta-mercaptoethanol. Samples were then transferred to polyvinylidene difluoride membranes (Bio-Rad) or stained by Coomassie brilliant blue. Membranes were incubated with rabbit polyclonal anti-CheY at a 1:1,500 dilution in milk and followed by incubation with horseradish peroxidase-conjugated goat anti-rabbit antibodies (Santa Cruz Biotech) at a 1:1,500 dilution (66). Luminescent blots were then visualized by BioMax light film (Kodak). Gels stained by Coomassie were visualized by Bio-Rad ChemiDoc MP.

### Soft agar migration assay

Overnight *H. pylori* cultures were diluted with BB10 to OD_600_ of 0.15. Two microliters of cells was inoculated in plates composed of Brucella Broth, 2.5% HI-FBS, and 0.35% (wt/vol) of agar (Bacto) using a pipette tip. The soft agar plates were cultured under microaerobic conditions. The diameter of each colony was measured after 4 days.

### Analysis of swimming behavior

Cells were cultured overnight with the BB10 medium as described above and were diluted into fresh BB10 to achieve an OD_600_ of 0.15. The diluted cells were incubated with 200 rpm shaking in microaerobic conditions at 37°C for 2 hours before being used to record the swimming behavior at 400× magnification with the Nikon Eclipse E600 phase-contrast microscope and recorded by a Hamamatsu C4742-95 digital camera with MicroManager (82). ImageJ was used to analyze the trajectory with the TrackMate plugin, with the frequency of direction changes counted within a 3-s continuous swimming trajectory.

### Statistical analysis

Data were analyzed statistically using SPSS software (version 20, IBM Corp., Armonk, New York) by the application of Student’s *t*-test or one-way ANOVA with Tukey’s test. *P* < 0.05 or <0.01 were considered statistically significant.

## SUPPLEMENTAL MATERIAL

Supplemental Figures mbio.00440-24-s0001.docx Figures S1 and S2.

ASM does not own the copyrights to Supplemental Material that may be linked to, or accessed through, an article. The authors have granted ASM a non-exclusive, world-wide license to publish the Supplemental Material files. Please contact the corresponding author directly for reuse.
