# Supplementary material for: Counterclockwise rotation of the flagellum promotes biofilm initiation in Helicobacter pylori
Source: mBio. 2024 May 3;15(6):e00440-24. doi: 10.1128/mbio.00440-24 (PMC11237671; doi:10.1128/mbio.00440-24)
Supplement: Supplemental Figures — Figures S1 and S2. [file mbio.00440-24-s0001.docx]

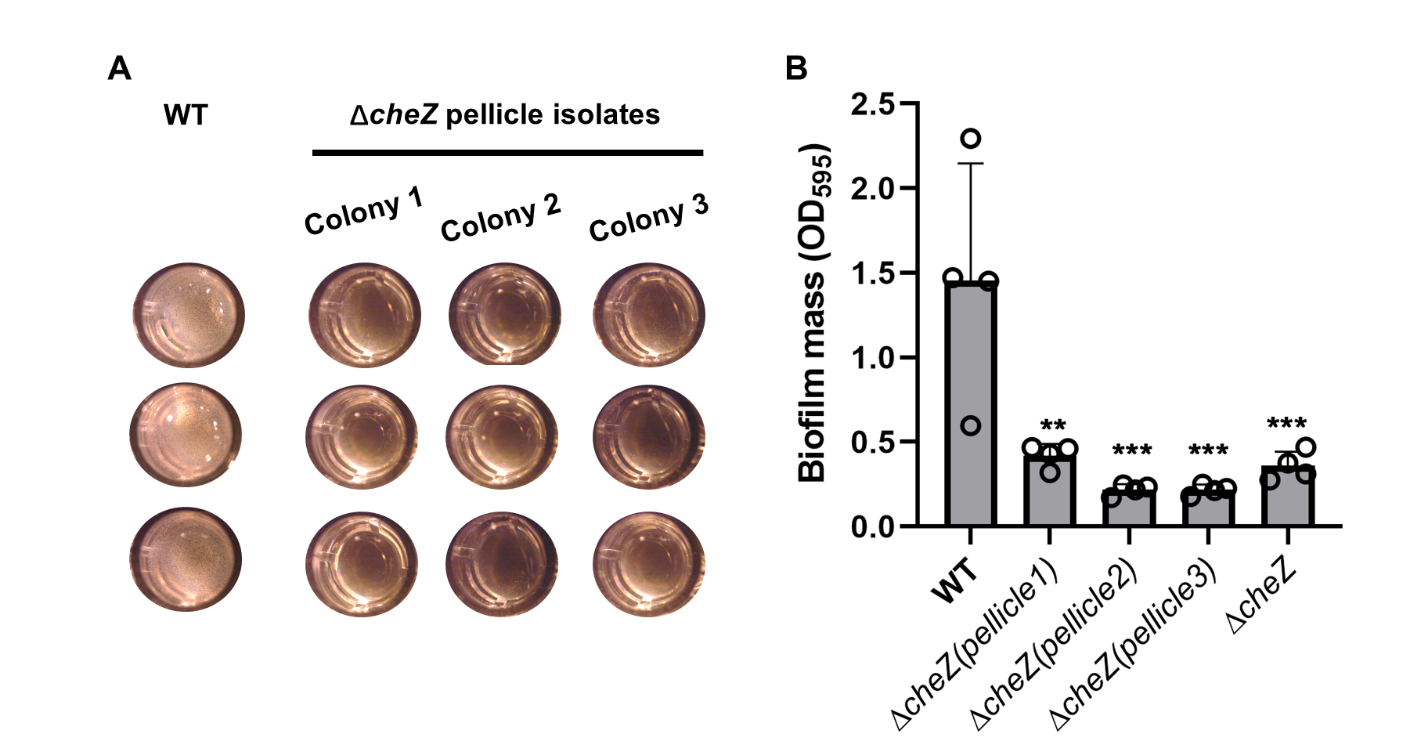


### Figure S1. Pellicle and biofilm formation of Δ*cheZ* colonies isolated from day 4 Δ*cheZ* pellicles.

*H. pylori* Δ*cheZ* mutants were cultured in 96-well under static condition for 4 days to form pellicles. Three independent colonies were isolated from 4-day old Δ*cheZ* pellicles. (A) WT and three Δ*cheZ(pellicle)* colonies were cultured in 96-well under static conditions for 1 day to form pellicles. (B) Biofilm formation of WT, three Δ*cheZ(*pellicle*)* colonies, and Δ*cheZ* mutant for 1 day. Data shown are the means ± SDs from at least three independent experiments. Statistical analyses were performed using ANOVA (**, p<0.01; ***, p<0.001; ****, p<0.0001), with asterisks indicating comparison to WT.


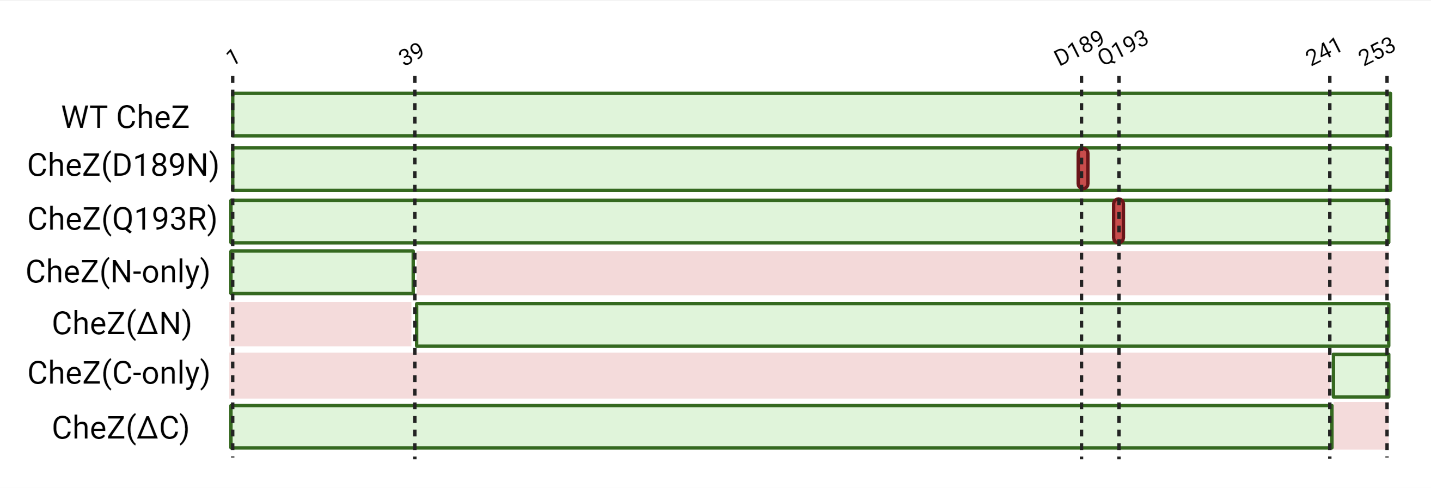


### Figure S2. Diagram of the *H. pylori* CheZ truncated proteins.

WT CheZ is depicted in light green on the top line, with the amino acid residue numbers indicated by vertical dashed lines. D189 and Q193 are CheZ active site residues (63). In truncated variants (rows 2-6), deleted regions are shown with light red. The site substitution mutants D189N and Q193R are shown with red boxes. The region retained in each CheZ protein is shown in light green.
